# Feeding Practices and Nutritional Status of Last-Born Children Aged 0–23 Months in India: Evidence From the National Family Health Survey, 2019-21

**DOI:** 10.7759/cureus.84619

**Published:** 2025-05-22

**Authors:** Abhishek Singh, Prashant Singh, Saurabh Kashyap, Rizwana Bano

**Affiliations:** 1 Community Medicine and Public Health, King George's Medical University, Lucknow, IND; 2 Quality Assurance, UP (Uttar Pradesh) National Health Mission, Lucknow, IND

**Keywords:** biodemographic factors, child undernutrition, exclusive breast feeding, feeding practices, socioeconomic factors

## Abstract

Introduction

Child-feeding practices in the first two years of life play an important role in children's overall growth. India has started many countrywide initiatives to encourage improved nutrition and care for women and children. However, important obstacles like limited health system capacity and behavioral resistance still must be addressed. Therefore, this study aims to investigate how child-feeding practices affect the nutritional status of children in India.

Materials and methods

The publicly available data used in this study were obtained from the latest round of the National Family Health Survey (NFHS-5) conducted in 2019-2021. The Z-score method of anthropometric indicator weight-for-age (underweight) was used to assess the nutritional status of last-born children aged 0-23 months. Child-feeding, biodemographic, and socioeconomic variables were used as the risk factors for low weight-for-age. Univariate, bivariate, and multivariate statistical analyses were carried out to fulfill the study's objectives. The regression results were reported by unadjusted odds ratio (UOR) and adjusted odds ratio (AOR) with 95% CI and p-value.

Results

Children who started breastfeeding more than an hour after delivery had an 8% (95% CI: 1.03 to 1.09) higher chance of being underweight than children who started breastfeeding within an hour. Children who were breastfed for more than 18 months had a 62% (95% CI: 1.54 to 1.70) higher risk of being underweight than children who were breastfed for less than six months. The likelihood of being underweight was lower among female children (OR=0.77; 95% CI: 0.75 to 0.80) as compared with male children. Children from the richest wealth index were 62% (95% CI: 0.36 to 0.39) less likely to be underweight than those from the poorest wealth index. Children of mothers with an education of high school and above had a (OR: 0.51, 95% CI: 0.36 to 0.39) lower risk of being underweight compared to children of mothers with no education. The predicted probability of low weight-for-age among children was also greater (0.58, 95% CI: 0.56 to 0.60) if breastfeeding began more than an hour after delivery than within an hour of birth (0.57, 95% CI: 0.56 to 0.60).

Conclusion

Our study highlights the complex interplay of child-feeding practices, biodemographic, and socioeconomic factors influencing low weight-for-age among Indian children aged 0-23 months. The key findings emphasize the importance of the initiation of breastfeeding within one hour in preventing undernutrition. Socioeconomic disparities, particularly among marginalized communities, significantly increase the risk of children being underweight. The unexpected associations with prelacteal and bottle feeding suggest the need for deeper exploration into cultural and contextual feeding practices. Strengthening maternal education and improving access to nutrition and healthcare, especially for disadvantaged groups, can play a crucial role in addressing underweight prevalence among children in India.

## Introduction

Early life good nutrition sets the basis for a child's physical, cognitive, and emotional development. But many kids in India begin life at a disadvantage. The most recent National Family Health Survey (NFHS-5, 2019-21) shows a troubling trend of more than 32% of children under five are underweight, over one-fifth are wasted, and over one-third are stunted [1]. These figures are not only statistics, but they also represent millions of young lives impacted by undernutrition, usually because of poor feeding habits. A child’s feeding in the first two years shapes their lives both now and later. Breastfeeding within one hour of birth, exclusive breastfeeding for the first six months, and the progressive introduction of safe and varied complementary foods thereafter, all while continuing to breastfeed for up to two years or longer, contribute to the healthy growth of children [2]. Many Indian families, meanwhile, find these suggestions sometimes beyond reach. About 42% of Indian neonates are breastfed within one hour of birth; exclusive breastfeeding rates hover just above 63% [3].

Often, the introduction of complementary foods, a major turning point in a baby’s growth, is delayed or mishandled. In India, less than half of babies six to eight months old get timely solid or semi-solid meals [4]. Many youngsters between the ages of six and 23 months are not eating a varied enough diet to satisfy their nutritional demands [5]. Lack of diversity in the diet causes hidden hunger-micronutrient deficits compromising development, learning, and immunological function. Social and economic realities also impact how children are fed; it is not only biology. Families with lower incomes and mothers with inadequate education often struggle more to provide ideal nutrition [6]. Early weaning, usage of prelacteal meals, and feeding practices that do more damage than benefit can also result from cultural customs, false information, and lack of assistance from health services [7].

Realizing the seriousness of this problem, India has started many countrywide initiatives meant to encourage improved nutrition and care for women and children, including POSHAN (Prime Minister's Overarching Scheme for Holistic Nourishment) Abhiyaan and the Integrated Child Development Services (ICDS) [8]. Though actual execution sometimes differs, important obstacles like limited health system capacity and behavioral resistance still must be addressed [9,10].

Therefore, this study aims to go deeper considering this background, we investigate how child-feeding practices affect Indian children’s nutritional status. Doing so will help us to find not only what is effective but also where more work must be redirected to guarantee every kid a good beginning to life.

## Materials and methods

Data and study population

The nationally representative secondary data used in this study were obtained from the cross-sectional survey of the National Family Health Survey (NFHS-5) conducted in 2019-2021 [11]. The two-stage stratified sampling was used by the International Institute for Population Sciences (IIPS), under the stewardship of the Ministry of Health and Family Welfare, Government of India, New Delhi. All living last-born children (n = 87,267) aged 0-23 months who participated in NFHS-5 were the primary sampling units and hence enrolled in the study. The sampling frame for the selection of primary sampling units was the 2011 census data. The NFHS-5 collected information on variables, namely women’s background characteristics, household economic status, healthcare-seeking behavior, and complete birth histories of women, including each child’s date of birth, sex, and nutritional status [11].

Outcome variable

In this study, the Z-score method of anthropometric indicator weight-for-age (underweight) was used to assess the nutritional status of last-born children aged 0-23 months. To assess the nutritional status of the whole population, the most reliable and credible indicator is the weight-for-age index [12]. The anthropometric indicator weight-for-age of child nutritional status was categorized into two categories: underweight (< -2.0 Z-score) and not underweight (>= 2.0 Z-score).

Explanatory variables

Child-Feeding Variables

Child-feeding variables were treated as the major explanatory variables in this study. It included the initiation of breastfeeding, receiving prelacteal feed, breastfeeding status, duration of breastfeeding, and drinking from a bottle with a nipple. First, initiation of breastfeeding was divided into two categories: within one hour and more than one hour. Further, prelacteal feeding, referred to as children given something other than breast milk during the first three days of life, was divided into two categories: receiving a prelacteal feed or not receiving a prelacteal feed. The composite variable of breastfeeding and child-feeding practices was generated into six categories: not breastfed, exclusively breastfed for six months, breastfed and plain water, breastfed and non-milk liquids, breastfed and other milk, and breastfed and complementary food (solids and semi-solids). Duration of breastfeeding was grouped into four categories: less than six months, six months to one year, 13 months to 18 months, and 18+ months. The bottle feeding of children was divided into two categories: drinking from a bottle with a nipple and not drinking from a bottle with a nipple.

Biodemographic Variables

The biodemographic variables used in the study were: age of child in months (<6, 6-11, 12-17, and 18-23), sex of the child (male and female), age of mother at the birth of the child (less than 18 years, 18-24 years, 25-34 years, and 35-49 years), composite variable of birth order and interval (first order, order 2-3 and <24 months, order 2-3 and >=24 months, order 3+ and <24 months, and order 3+ and >=24 months), and mother’s BMI (underweight, normal, overweight/obese).

Socioeconomic Variables

The socioeconomic variables used in the study were: place of residence (urban and rural), religion (Hindu and Others), caste (scheduled tribes and scheduled castes (SC/STs), other backward castes (OBCs), and others), household wealth index (poorest, poorer, middle, richer, and richest), and mother’s education (no education, up to primary level complete, up to secondary level complete, and high school and above).

Statistical analysis

Univariate, bivariate, and multivariate statistical analyses were carried out to fulfill the study's objectives. At the univariate level, descriptive statistics were employed to understand the distribution of child-feeding practices, and the biodemographic, and socioeconomic characteristics of the study sample. The bivariate analysis included the estimation of the prevalence of last-born underweight children aged 0-23 months by child-feeding, biodemographic, and socioeconomic variables. Pearson’s chi-square test was used with p < 0.05 to check the significance difference between outcome and independent variables. The sample weight was used for the estimation of the percentage distribution of variables.

The outcome variable in our analysis was low weight-for-age (underweight) (no/yes). Owing to the binary nature of the outcome variable, the bivariate and multivariate binary logistic regression models were performed to investigate the effect of child-feeding variables on the outcome variable. The regression results were reported by unadjusted odds ratio (UOR) and adjusted odds ratio (AOR) with 95% CI and p-value. Besides, predicted probabilities [13] were also estimated for child-feeding practice variables to evaluate the behavior of child-feeding variables on the outcome variable. All statistical analyses were carried out using the statistical software R version 4.4.1 (R Foundation for Statistical Computing, Vienna, Austria) [14], and results were interpreted as statistically significant at a p-value of < 0.05.

Ethics

This study used the NFHS-5 data available in the public domain for use by researchers, thus, no ethical approval is required for this study.

## Results

Distribution of study sample by child-feeding, biodemographic, and socioeconomic variables

Table 1 shows the percentage distribution of last-born children aged 0-23 months based on child-feeding, biodemographic, and socioeconomic factors. For child-feeding habits, just 43.2% of kids were breastfed within one hour of delivery, and 56.8% started breastfeeding after one hour. Most (84.5%) had no prelacteal feeding. While 47.8% were being breastfed with supplementary foods, 21.8% of kids reported exclusive breastfeeding. Of the children, 29.7% were breastfed for less than six months, and 21.2% drank bottle milk with a nipple. With the greatest percentage being under six months (26.6%), the sample was evenly spread across the age categories of children. Of the sample, 51.9% were males. At the time of delivery, most women were between 18 and 24 years old (52.1%) or 25 to 34 years old (42.3%). First-born kids made up almost 39.4%, and second- or third-birth-order kids made up 36.7%, with an interpregnancy gap of at least 24 months.

**Table 1:** Percent distribution of last-born children aged 0-23 months by child-feeding, biodemographic and socioeconomic variables in India, NFHS-5 (2019-21) SC/ST: Scheduled Castes/Scheduled Tribes, OBC: Other Backward Classes, NFHS: National Family Health Survey

Variables	%	N
Child-Feeding Variables		
Initiation of breastfeeding		
Within 1 hour	43.2	36143
More than 1 hour	56.8	47541
Received a prelacteal feed		
No	84.5	70709
Yes	15.5	12976
Breastfeeding status		
Not breastfed	15.2	13220
Exclusively breastfed for 6 months	21.8	18992
Breastfed & plain water	7.4	6418
Breastfed & non-milk liquids	2.0	1703
Breastfed & other milk	6.0	5202
Breastfed & complementary foods	47.8	41733
Duration of breastfeeding		
Less than 6 months	29.7	24802
6 months to 1 year	33.0	27589
13 months to 18 months	23.0	19187
18+ months	14.3	12019
Drank from a bottle with a nipple		
No	78.8	66705
Yes	21.2	17901
Biodemographic Variables		
Age of child (in months)		
<6	26.6	23232
6-11	25.6	22333
12-17	25.4	22167
18-23	22.4	19535
Sex of the child		
Male	51.9	45305
Female	48.1	41963
Age of mother at birth of the child		
Less than 18 years	2.0	1761
18-24 years	52.1	45444
25-34 years	42.3	36878
35-49 years	3.7	3184
Birth order & interval		
First order	39.4	34199
Order 2-3 &<24 months	12.3	10722
Order 2-3 &>=24 months	36.7	31927
Order 3+ &<24 months	2.9	2489
Order 3+ & 24 >= months	8.7	7564
Mother’s BMI		
Underweight	21.3	17923
Normal	62.1	52328
Overweight/obese	16.6	13974
Socioeconomic Variables		
Place of residence		
Urban	25.9	22606
Rural	74.1	64661
Religion		
Hindu	79.4	69299
Others	20.6	17969
Caste		
SC/STs	35.4	29224
OBCs	46.0	37948
Others	18.6	15325
Wealth index		
Poorest	24.1	21001
Poorer	21.6	18827
Middle	19.9	17319
Richer	18.6	16269
Richest	15.9	13852
Mother’s education		
No education	18.9	16481
Up to primary level complete	17.7	15459
Up to secondary level complete	19.1	16694
High school and above	44.3	38634
Number of cases	100	87267

Association of child-feeding, biodemographic, and socioeconomic variables with nutritional status of last-born children aged 0-23 months

Table 2 shows the bivariate relationship between underweight status (based on weight-for-age) and child-feeding, biodemographic, and socioeconomic factors. Every relationship was statistically significant (p < 0.001). Underweight children (43.4%) were somewhat more likely to start breastfeeding early, within one hour than non-underweight children (42.9%). Of those who did not get prelacteal meals, 85.4% were underweight compared to 83.2%. Non-underweight kids (22.7%) had somewhat more exclusive breastfeeding than underweight (21.9%). Of underweight children, 51.0% were still breastfed while consuming supplemental foods. Compared to their non-underweight counterparts, children who were breastfed for more than 18 months (17%) had a higher prevalence of underweight, whereas those who were breastfed for less than six months (27.3%) had a lower prevalence. Non-underweight kids (24%) were more likely to bottle-feed than underweight children (19.3%). Among biodemographic factors, the prevalence of underweight children was greater among older youngsters (25.1% in the 18-23 months age group). In comparison to males being underweight (54.0%), female children have a lower percentage of being underweight (46.0%). Children of women under 18 years (2.4%) and those of greater birth order with short interpregnancy intervals were similarly more likely to be underweight. Maternal nutritional status revealed a clear link with low weight-for-age. Of underweight children, 25.9% had underweight mothers compared to 15.1% among non-underweight children. Rural areas (76.6%) had a greater underweight than urban areas (23.4%). Underweight children were more likely to be OBCs (46.0%) and from the lowest wealth quintile (28.5%). Underweight status was inversely related to mothers' educational level; just 39.1% of underweight children had mothers educated up to high school and higher, vis-à-vis, 51.9% among non-underweight children.

**Table 2:** Association of last-born underweight children aged 0-23 months by child-feeding, biodemographic and socioeconomic variables in India, NFHS-5 (2019-21) * p-value is calculated by the Pearson's Chi-square test SC/ST: Scheduled Castes/Scheduled Tribes, OBC: Other Backward Classes, NFHS: National Family Health Survey

Variables	Underweight (weight-for-age)			
	No	Yes	χ^2^	p-value*
Child-Feeding Variables				
Initiation of breastfeeding				
Within 1 hour	42.9	43.4	13.21	0.000
More than 1 hour	57.1	56.6		
Received prelacteal feed				
No	83.2	85.4	80.46	0.000
Yes	16.8	14.6		
Breastfeeding status				
Not breastfed	13.7	11.6	87.97	0.000
Exclusively breastfed	22.7	21.9		
Breastfed & plain water	7.5	7.6		
Breastfed & non-milk liquids	2.1	1.9		
Breastfed & other milk	6.1	6.0		
Breastfed & complementary foods	47.9	51.0		
Duration of breastfeeding				
Less than 6 months	30.1	27.3	565.42	0.000
6 months to 1 year	35.8	31.7		
13 months to 18 months	22.7	24.1		
18+ months	11.4	17.0		
Drank from a bottle with a nipple				
No	76.0	80.7	234.83	0.000
Yes	24.0	19.3		
Biodemographic Variables				
Age of child (in months)				
<6	27.3	25.3	437.27	0.000
6-11	27.7	24.2		
12-17	25.7	25.4		
18-23	19.3	25.1		
Sex of the child				
Male	48.5	54.0	320.17	0.000
Female	51.5	46.0		
Age of mother at birth of the child				
Less than 18 years	1.6	2.4	148.09	0.000
18-24 years	50.8	53.4		
25-34 years	44.0	40.7		
35-49 years	3.5	3.6		
Birth order & interval				
First order	41.9	37.5	339.83	0.000
Order 2-3 &<24 months	11.2	13.2		
Order 2-3 &>=24 months	37.7	36.6		
Order 3+ &<24 months	2.1	3.2		
Order 3+ & 24 >= months	7.1	9.6		
Mother’s BMI				
Underweight	15.1	25.9	2100.00	0.000
Normal	63.3	61.1		
Overweight/obese	1.6	13.0		
Socioeconomic Variables				
Place of residence				
Urban	28.4	23.4	227.81	0.000
Rural	71.6	76.6		
Religion				
Hindu	79.0	80.2	361.47	0.000
Others	21.0	19.8		
Caste				
SC/STs	31.3	38.3	386.20	0.000
OBCs	46.7	45.5		
Others	22.0	16.2		
Wealth index				
Poorest	17.5	28.5	2000.00	0.000
Poorer	19.5	23.3		
Middle	20.5	19.8		
Richer	21.5	16.5		
Richest	21.0	11.9		
Mother’s education				
No education	14.7	21.3	1300.00	0.000
Up to primary level complete	15.1	19.4		
Up to secondary level complete	18.3	20.2		
High school and above	51.9	39.1		
Number of cases	32120	47134		

Bivariate and multivariate binary logistic regression analysis of underweight children

The four binary logistic regression models were estimated to assess the risk of being underweight associated with child-feeding practices, biodemographic, and socioeconomic factors. Table 3 shows unadjusted odds ratios (Model-1) based on bivariate binary logistic regression models, as well as adjusted odds ratios with child-feeding (Model-2), child-feeding and biodemographic (Model-3), and child-feeding, biodemographic, and socioeconomic (Model-4) variables using multivariate binary logistic regression models. The unadjusted odds ratios presented in Model-1 show that all the child-feeding, biodemographic, and socioeconomic factors are significantly associated with the low weight-for-age among last-born children aged 0-23 months.

**Table 3:** Binary logistic regression estimates of the effect of biodemographic, child-feeding and socioeconomic factors on prevalence of underweight children, India, NFHS-5 (2019-21) ref.: reference category, SC/ST: Scheduled Castes/Scheduled Tribes, OBC: Other Backward Classes, NFHS: National Family Health Survey

Variables	Underweight (weight-for-age)											
	Model-1 (unadjusted)			Model-2 child-feeding			Model-3 Model-2 + biodemographic			Model-4 Model-3 + socioeconomic		
	OR	p-value	95% CI	OR	p-value	95% CI	OR	p-value	95% CI	OR	p-value	95% CI
Child-Feeding Variables												
Initiation of breastfeeding												
Within 1 hour (ref.)	1.00			1.00			1.00			1.00		
More than 1 hour	1.06	0.000	1.03 to 1.09	1.08	0.000	1.05 to 1.12	1.07	0.000	1.04 to 1.10	1.03	0.084	1.00 to 1.06
Received prelacteal feed												
No (ref.)	1.00			1.00			1.00			1.00		
Yes	0.83	0.000	0.80 to 0.87	0.85	0.000	0.82 to 0.89	0.90	0.000	0.87 to 0.94	0.94	0.003	0.90 to 0.98
Breastfeeding status												
Not breastfed (ref.)	1.00			1.00			1.00			1.00		
Exclusively breastfed	1.15	0.000	1.08 to 1.21	1.08	0.010	1.02 to 1.15	1.18	0.000	1.09 to 1.28	1.11	0.010	1.03 to 1.20
Breastfed & plain water	1.18	0.000	1.10 to 1.26	1.08	0.042	1.00 to 1.16	1.14	0.003	1.04 to 1.24	1.07	0.148	0.98 to 1.16
Breastfed & non-milk liquids	1.11	0.067	0.99 to 1.25	0.98	0.766	0.87 to 1.10	1.06	0.354	0.94 to 1.20	1.04	0.580	0.91 to 1.18
Breastfed & other milk	1.18	0.000	1.09 to 1.27	1.16	0.000	1.07 to 1.26	1.24	0.000	1.13 to 1.35	1.17	0.001	1.07 to 1.29
Breastfed & complementary foods	1.22	0.000	1.16 to 1.29	1.01	0.824	0.95 to 1.06	1.07	0.060	1.00 to 1.14	1.03	0.413	0.96 to 1.10
Duration of breastfeeding												
Less than 6 months (ref.)	1.00			1.00			1.00			1.00		
6 months to 1 year	0.96	0.058	0.93 to 1.00	1.03	0.256	0.98 to 1.07	0.98	0.765	0.88 to 1.10	0.98	0.776	0.88 to 1.10
13 months to 18 months	1.18	0.000	1.13 to 1.23	1.27	0.000	1.20 to 1.33	1.11	0.090	0.98 to 1.25	1.11	0.087	0.98 to 1.26
18+ months	1.62	0.000	1.54 to 1.70	1.73	0.000	1.63 to 1.83	1.29	1.49	1.13 to 1.49	1.29	0.000	1.12 to 1.48
Drank from a bottle with a nipple												
No (ref.)	1.00			1.00			1.00			1.00		
Yes	0.77	0.000	0.74 to 0.80	0.79	0.000	0.76 to 0.82	0.84	0.000	0.80 to 0.87	0.89	0.000	0.86-0.93
Biodemographic Variables												
Age of child (in months												
<6 (ref.)	1.00						1.00			1.00		
6-11	0.94	0.006	0.91 to 0.98				0.99	0.898	0.88 to 1.12	0.98	0.699	0.87 to 1.10
12-17	1.07	0.001	1.03 to 1.12				1.06	0.370	0.93 to 1.20	1.04	0.559	0.92 to 1.18
18-23	1.44	0.000	1.38 to 1.51				1.30	0.000	1.14 to 1.49	1.27	0.001	1.11 to 1.46
Sex of the child												
Male (ref.)	1.00						1.00			1.00		
Female	0.77	0.000	0.75 to 0.80				0.77	0.000	0.75 to 0.80	0.76	0.000	0.74 to 0.79
Age of mother at birth of the child												
Less than 18 years (ref.)	1.00						1.00			1.00		
18-24 years	0.80	0.000	0.70 to 0.90				0.81	0.001	0.71 to 0.92	0.91	0.131	0.80-1.03
25-34 years	0.69	0.00	0.61 to 0.78				0.70	0.000	0.62 to 0.80	0.86	0.025	0.76-0.98
35-49 years	0.67	0.000	0.58 to 0.77				0.64	0.000	0.56 to 0.75	0.80	0.003	0.69-0.92
Birth order & interval												
First order (ref.)	1.00						1.00			1.00		
Order 2-3 &<24 months	1.33	0.000	1.27 to 1.40				1.33	0.000	1.26 to 1.40	1.20	0.000	1.14 to 1.27
Order 2-3 &>=24 months	1.07	0.000	1.04 to 1.11				1.15	0.000	1.10 to 1.19	1.03	0.176	0.99 to 1.07
Order 3+ &<24 months	1.64	0.000	1.49 to 1.80				1.74	0.000	1.58 to 1.92	1.28	0.000	1.16 to 1.42
Order 3+ & 24 >= months	1.38	0.000	1.31 to 1.46				1.54	0.000	1.45 to 1.64	1.13	0.000	1.05 to 1.20
Mother’s BMI												
Underweight (ref.)	1.00						1.00			1.00		
Normal	0.53	0.000	0.51 to 0.55				0.54	0.000	0.52 to 0.56	0.58	0.000	0.56 to 0.61
Overweight/obese	0.33	0.000	0.32 to 0.35				0.36	0.000	0.34 to 0.38	0.45	0.000	0.42 to 0.47
Socioeconomic Variables												
Place of residence												
Urban (ref.)	1.00									1.00		
Rural	1.31	0.000	1.26 to 1.36							0.88	0.000	0.84-0.92
Religion												
Hindu (ref.)	1.00									1.00		
Others	0.70	0.000	0.68 to 0.72							0.74	0.000	0.71-0.77
Caste												
SC/STs (ref.)	1.00									1.00		
OBCs	0.96	0.023	0.93 to 0.99							1.07	0.000	1.03-1.10
Others	0.67	0.000	0.64 to 0.69							0.91	0.000	0.87-0.95
Wealth index												
Poorest (ref.)	1.00									1.00		
Poorer	0.73	0.000	0.70 to 0.76							0.81	0.000	0.78 to 0.85
Middle	0.60	0.000	0.57 to 0.63							0.73	0.000	0.69 to 0.76
Richer	0.49	0.000	0.47 to 0.51							0.64	0.000	0.61 to 0.68
Richest	0.38	0.000	0.36 to 0.39							0.55	0.000	0.51 to 0.58
Mother’s education												
No education (ref.)	1.00									1.00		
Up to primary level complete	0.86	0.000	0.81 to 0.90							0.95	0.055	0.90 to 1.00
Up to secondary level complete	0.73	0.000	0.69 to 0.76							0.83	0.000	0.79 to 0.87
High school and above	0.51	0.000	0.49 to 0.54							0.72	0.000	0.68 to 0.75

According to Model-2, children who started breastfeeding more than an hour after delivery had an 8% (95% CI: 1.03 to 1.09) higher chance of being underweight than children who started breastfeeding within an hour. Compared to children who did not receive prelacteal feed, children who received it had a 17% (95% CI: 0.80 to 0.87) lower risk of being underweight. The category-specific pattern of breastfeeding status reveals a negative association between breastfeeding and the likelihood of being underweight in children. Children who were breastfed for more than 18 months had a 62% (95% CI: 1.54 to 1.70) higher risk of being underweight than children who were breastfed for less than six months. The likelihood of being underweight was lower among children who drank from a bottle with a nipple (OR=0.77; 95% CI: 0.74 to 0.80) as compared with those who did not drink from a bottle with a nipple.

Following the adjustment of biodemographic variables along with child-feeding factors in Model-3, the results indicate that child-feeding variables played a similar role as in the previous model. Among the biodemographic variables, children aged 18-23 months had a greater chance of being underweight (OR=1.30, 95% CI: 1.14 to 1.49) compared to children under six months old. The likelihood of being underweight was lower among female children (OR=0.77; 95% CI: 0.75 to 0.80) as compared with male children. The age-wise distribution of mothers points to a negative association between a child's likelihood of being underweight and the mother's increasing age at childbirth. On the other hand, the composite variable of birth order and birth interval reveals a positive association between an increase in birth order and an increasing or decreasing birth interval of the child, with the likelihood of being underweight among children. Children with birth order 3+ and interval < 24 months were at a 74.0% (95% CI: 1.58 to 1.92) higher risk of being underweight compared to those with first birth order. Children of normal-weight mothers were less likely (OR: 0.53, 95% CI: 0.51 to 0.55) to be underweight than those of underweight mothers.

Model-4, adjusting for socioeconomic variables along with child-feeding and biodemographic variables, reveals that the child-feeding and biodemographic variables exhibited comparable behavior to the earlier models. Among the socioeconomic variables, compared to children from urban backgrounds, children from rural backgrounds had a lower likelihood of being underweight (OR: 0.88, 95% CI: 0.84-0.92). Children of other religions had a lower risk of being underweight (OR: 0.70, 95% CI: 0.68-0.72) than Hindu children. Similarly, compared to children from the SC/STs community, children from other castes had a lower likelihood of being underweight (OR: 0.67, 95% CI: 0.64-0.69). The wealth index distribution indicates a negative association between a child's risk of being underweight and the children from the lowest to the highest wealth index. Children from the richest wealth index were 62.0% (95% CI: 0.36 to 0.39) less likely to be underweight than those from the poorest wealth index. Similarly, the distribution of mothers’ education indicates a negative association between a child's likelihood of being underweight and the children of mothers with no education to high school and above. Children of mothers with an education of high school and above had a (OR: 0.51, 95% CI: 0.36 to 0.39) lower risk of being underweight compared to children of mothers with no education.

Predicted probabilities for low weight-for-age by child-feeding variables

**Figure 1:**
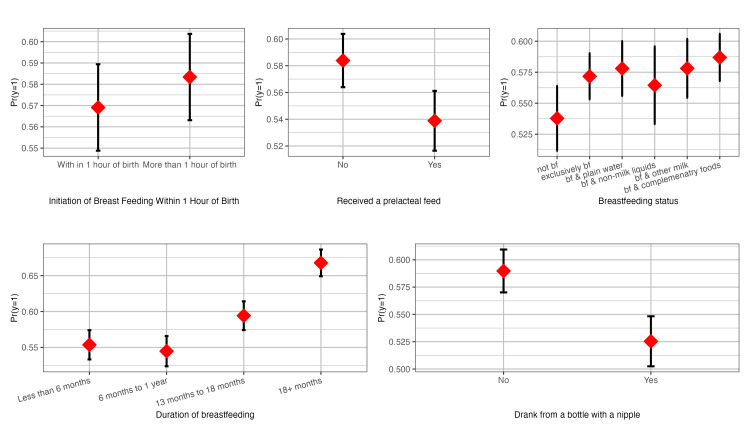
Predicted probabilities for low weight-for-age by child-feeding variables The image is an original creation. Image credits: Abhishek Singh

The estimated predicted probabilities of low weight-for-age among last-born children by the initiation of breastfeeding, prelacteal feeding, breastfeeding status, duration of breastfeeding, and drinking from a bottle with a nipple are presented in Figure 1. The predicted probability of low weight-for-age among children was somewhat greater (0.58, 95% CI: 0.56 to 0.60) if breastfeeding began more than an hour after delivery than within an hour of birth (0.57, 95% CI: 0.56 to 0.60). Children who did not receive a prelacteal feed had a greater predicted probability of having low weight-for-age (0.58, 95% CI: 0.56 to 0.60) than children who received a prelacteal feed (0.53, 95% CI: 0.51 to 0.56). Compared to exclusive breastfeeding and breastfeeding in combination, the predicted probability of low weight-for-age in children was marginally lower (0.53, 95% CI: 0.51 to 0.56) for children who were not breastfed. Children who were breastfed for a longer period had a higher predicted probability of being underweight for their age than those who were breastfed for a shorter period. The predicted probability for low weight-for-age among children was lower (0.53, 95% CI: 0.50 to 0.55) who drank from a bottle with a nipple than for children who did not drink from a bottle with a nipple (0.58, 95% CI: 0.57 to 0.61).

## Discussion

Using the NFHS-5 dataset (2019-21), this study looked at how child-feeding habits, biodemographic, and socioeconomic factors are related to the low weight-for-age children in India between the ages of 0 and 23 months. The results show that low weight-for-age is caused by a complex relationship of child-feeding practices with biodemographic and deep-seated socioeconomic factors.

Our study showed that starting breastfeeding later (after one hour of birth) was significantly linked to a higher prevalence of underweight. This is in line with earlier research that shows starting breastfeeding early is important for an infant's health and survival [15,16]. Notably, exclusive breastfeeding did not show a consistent protective link in the fully adjusted model. This could mean that there are problems with when or how well the complementary feeding starts [17]. The study found that children who received a prelacteal feed had a slightly lower chance of being underweight, which goes against what is usually suggested. This might be because of bias in the reports or cultural practices where prelacteal feeds are given along with the best breastfeeding support [18]. Also, children who were fed through bottles seemed less likely to be underweight, which could be a sign of a higher socioeconomic level or better literacy among the mothers [19]. The age of the child was a strong predictor of being underweight, with the biggest risk seen in children between the ages of 18 and 23 months.

This is in line with other research that has shown how vulnerable children are during the weaning transition [20]. Girls were much less likely to be underweight than boys. This finding is consistent with the previous study [21]. The age of the mother at birth was linked to a lower risk of low weight-for-age, especially for women aged 25 to 34. Teenage motherhood (less than 18 years) has always been linked to poor birth and growth results because the mother is biologically immature and has less access to care [22,23]. Higher birth order and shorter birth intervals were also strongly linked to being underweight. This supports the idea that having a lot of babies so quickly can hurt both mothers and their children's nutrition [24]. Also, there was a strong link between the mother's BMI and the child's nutritional state. Children born to underweight mothers had a much higher chance of being underweight. This supports what other studies [25,26] have found about how malnutrition can be passed down from parent to child.

Socioeconomic differences also turned out to be the significant risk factors of low weight-for-age among children. Children from SC/STs groups, low-income families, and rural areas were at a much higher risk. This fits with past research that showed that marginalized groups often have bad sanitation, limited access to health care, and trouble getting enough food [27,28]. Even after income and residence were considered, a mother's level of education was found to be a strong predictor of not being overweight. This result backs up the well-known fact that mothers who can read and write are more likely to take care of their children's health and be aware of good nutrition [29]. So, programs that work to educate and support women could have long-lasting effects on their children's health.

## Conclusions

Our study highlights the complex interplay of child-feeding practices, biodemographic, and socioeconomic factors influencing low weight-for-age among Indian children aged 0-23 months. The key findings emphasize the importance of the initiation of breastfeeding within one hour in preventing undernutrition. Socioeconomic disparities, particularly among marginalized communities, significantly increase the risk of underweight children. The unexpected associations with prelacteal and bottle feeding suggest the need for deeper exploration into cultural and contextual feeding practices. Strengthening maternal education and improving access to nutrition and healthcare, especially for disadvantaged groups, can play a crucial role in addressing underweight prevalence among children in India.
